# Crystal structure of 2-cyano-*N*′-(cyclo­hexyl­idene)acetohydrazide

**DOI:** 10.1107/S1600536814009350

**Published:** 2014-08-01

**Authors:** William T. A. Harrison, Ola K. Al-Sakka, Daisy H. Fleita, Amina Saleh, Sara Salem

**Affiliations:** a Department of Chemistry, University of Aberdeen, Meston Walk, Aberdeen AB24 3UE, Scotland; b Department of Chemistry, American University in Cairo, PO Box 74, New Cairo 11835, Egypt; c Institute of Chemistry and Chemical Engineering, École Polytechnique Fédérale de Lausanne, Station 6, CH-1015 Lausanne, Switzerland

**Keywords:** crystal structure, hydrazide, cyclo­hexyl­idene, inversion dimer

## Abstract

In the title compound, C_9_H_13_N_3_O, the cyclo­hexyl­idene ring adopts a chair conformation and the bond-angle sum at the C atom linked to the N atom is 359.6°. The cyano­acetohydrazide grouping is close to planar (r.m.s. deviation for the non-H atoms = 0.031 Å) and subtends a dihedral angle of 64.08 (4)° with the four C atoms forming the seat of the chair. The C=O and N—H groups are in a *syn* conformation (O—C—N—H = −5°). In the crystal, inversion dimers linked by pairs of N—H⋯O hydrogen bonds generate *R*_2_^2^(8) loops; this dimer linkage is reinforced by a pair of C—H⋯O inter­actions, which generate *R*_2_^2^(14) loops. The dimers are linked by C—H⋯N_c_ (c = cyanide) inter­actions into [100] ladders, which feature *C*(4) chains and *R*_4_^4^(20) loops.

## Related literature   

For background to the role of hydrazides as potential anti-cancer agents, see: Sechi *et al.* (2008 ▶); Manivel *et al.* (2009 ▶); Mohareb *et al.* (2011 ▶).
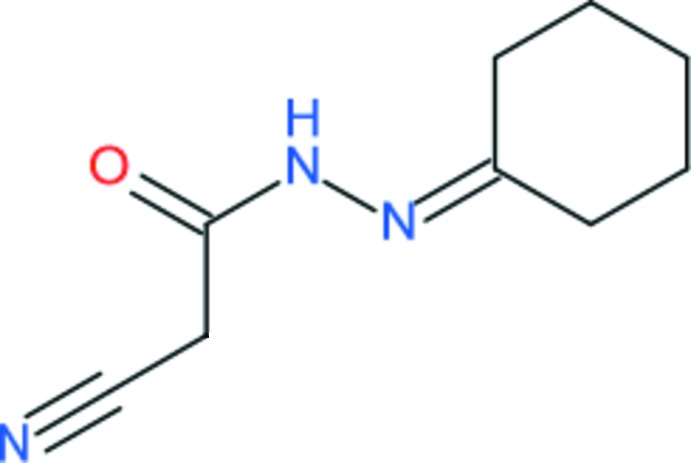


## Experimental   

### Crystal data  

C_9_H_13_N_3_O
*M* *_r_* = 179.22
Triclinic, 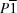

*a* = 4.8420 (2) Å
*b* = 9.7407 (7) Å
*c* = 10.7071 (8) Å
α = 73.917 (9)°
β = 82.819 (10)°
γ = 75.980 (9)°
*V* = 469.87 (5) Å^3^
*Z* = 2
Mo *K*α radiation
μ = 0.09 mm^−1^
*T* = 100 K
0.13 × 0.12 × 0.04 mm

### Data collection  

Rigaku Mercury CCD diffractometer
6176 measured reflections
2136 independent reflections
1789 reflections with *I* > 2σ(*I*)
*R*_int_ = 0.024

### Refinement  

*R*[*F* ^2^ > 2σ(*F* ^2^)] = 0.035
*wR*(*F* ^2^) = 0.101
*S* = 1.08
2136 reflections
121 parameters
H atoms treated by a mixture of independent and constrained refinement
Δρ_max_ = 0.29 e Å^−3^
Δρ_min_ = −0.19 e Å^−3^

### 

Data collection: *CrystalClear* (Rigaku, 2012 ▶); cell refinement: *CrystalClear*; data reduction: *CrystalClear*; program(s) used to solve structure: *SHELXS97* (Sheldrick, 2008 ▶); program(s) used to refine structure: *SHELXL97* (Sheldrick, 2008 ▶); molecular graphics: *ORTEP-3 for Windows* (Farrugia, 2012 ▶); software used to prepare material for publication: *SHELXL97*.

## Supplementary Material

Crystal structure: contains datablock(s) I, New_Global_Publ_Block. DOI: 10.1107/S1600536814009350/su0002sup1.cif

Structure factors: contains datablock(s) I. DOI: 10.1107/S1600536814009350/su0002Isup2.hkl

Supporting information file. DOI: 10.1107/S1600536814009350/su0002Isup3.cml

**Fig. 1.**
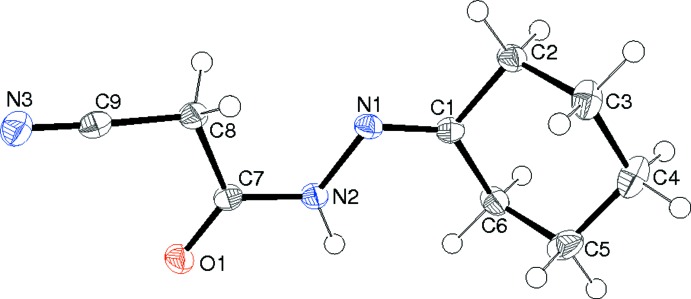
The molecular structure of the title compound showing 50% displacement ellipsoids.

. DOI: 10.1107/S1600536814009350/su0002fig1.tif
The mol­ecular structure of the title compound showing 50% displacement ellipsoids.

**Fig. 2.**
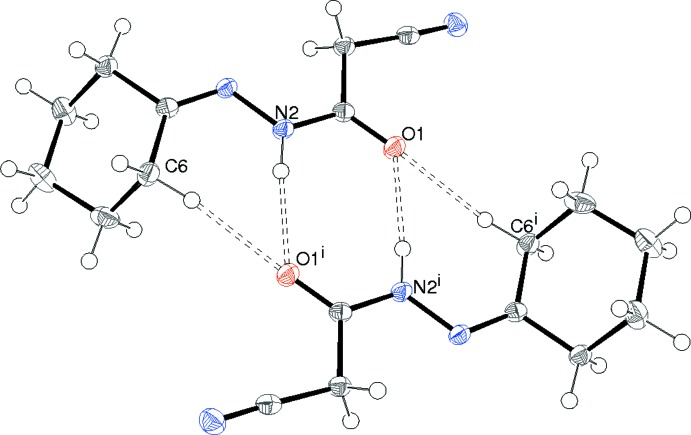
An inversion dimer in the crystal of the title compound, with N—H···O and C—H···O hydrogen bonds indicated by double-dashed lines. Symmetry code: (i) –x, 1–y, –z.

. DOI: 10.1107/S1600536814009350/su0002fig2.tif
An inversion dimer in the crystal of the title compound, with N—H⋯O and C—H⋯O hydrogen bonds indicated by double-dashed lines. Symmetry code: (i) –x, 1–y, –z.

**Fig. 3.**
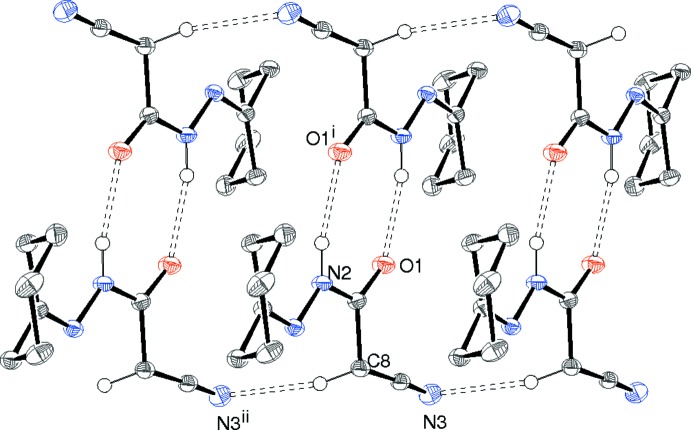
Part of a [100] double chain in the crystal of the title compound, with hydrogen bonds indicated by double-dashed lines. Symmetry codes: (i) –x, 1–y, –z; (ii) 1 + x, y, z.

. DOI: 10.1107/S1600536814009350/su0002fig3.tif
Part of a [100] double chain in the crystal of the title compound, with hydrogen bonds indicated by double-dashed lines. Symmetry codes: (i) –x, 1–y, –z; (ii) 1+x, y, z.

CCDC reference: 1004279

Additional supporting information:  crystallographic information; 3D view; checkCIF report

## Figures and Tables

**Table 1:** Hydrogen-bond geometry (Å, °)

*D*—H⋯*A*	*D*—H	H⋯*A*	*D*⋯*A*	*D*—H⋯*A*
N2—H1⋯O1^i^	0.900 (14)	2.052 (15)	2.9399 (12)	168.4 (11)
C6—H6*B*⋯O1^i^	0.99	2.32	3.2736 (13)	161
C8—H8*B*⋯N3^ii^	0.99	2.41	3.3783 (14)	165

Symmetry codes: (i) 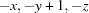; (ii) 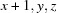.

## supplementary crystallographic information

### S1. Experimental

Cyclohexanone (0.98 g, 0.01 mol) was added to a solution of cyanoacetylhydrazine (0.99 g, 0.01 mol) in 1,4-dioxane (20 ml). The mixture was heated under reflux for 2 h and then poured into a beaker containing an ice/water mixture: the solid product was collected by filtration. Yellow slabs of the title compound were obtained by slow evaporation of an ethanol solution.

### S2. Refinement

The N-bound H atom was located in a difference map and its position was freely refined. The C-bound H atoms were placed in idealized locations (C—H = 0.99 Å) and refined as riding atoms. The constraint *U*_iso_(H) = 1.2*U*_eq_(carrier) was applied in all cases.

### Crystal data

**Table:**

C_9_H_13_N_3_O	*Z* = 2
*M**_r_* = 179.22	*F*(000) = 192
Triclinic, *P*1	*D*_x_ = 1.267 Mg m^−^^3^
Hall symbol: -P 1	Mo *K*α radiation, λ = 0.71073 Å
*a* = 4.8420 (2) Å	Cell parameters from 5790 reflections
*b* = 9.7407 (7) Å	θ = 2.6–27.5°
*c* = 10.7071 (8) Å	µ = 0.09 mm^−^^1^
α = 73.917 (9)°	*T* = 100 K
β = 82.819 (10)°	Cut slab, yellow
γ = 75.980 (9)°	0.13 × 0.12 × 0.04 mm
*V* = 469.87 (5) Å^3^	

### Data collection

**Table:**

Rigaku Mercury CCD diffractometer	1789 reflections with *I* > 2σ(*I*)
Radiation source: fine-focus sealed tube	*R*_int_ = 0.024
Graphite monochromator	θ_max_ = 27.5°, θ_min_ = 2.6°
ω scans	*h* = −6→5
6176 measured reflections	*k* = −12→11
2136 independent reflections	*l* = −13→13

### Refinement

**Table:**

Refinement on *F*^2^	Primary atom site location: structure-invariant direct methods
Least-squares matrix: full	Secondary atom site location: difference Fourier map
*R*[*F*^2^ > 2σ(*F*^2^)] = 0.035	Hydrogen site location: inferred from neighbouring sites
*wR*(*F*^2^) = 0.101	H atoms treated by a mixture of independent and constrained refinement
*S* = 1.08	*w* = 1/[σ^2^(*F*_o_^2^) + (0.0528*P*)^2^ + 0.0762*P*] where *P* = (*F*_o_^2^ + 2*F*_c_^2^)/3
2136 reflections	(Δ/σ)_max_ < 0.001
121 parameters	Δρ_max_ = 0.29 e Å^−^^3^
0 restraints	Δρ_min_ = −0.19 e Å^−^^3^

### Special details

**Table:**

Geometry. All e.s.d.'s (except the e.s.d. in the dihedral angle between two l.s. planes) are estimated using the full covariance matrix. The cell e.s.d.'s are taken into account individually in the estimation of e.s.d.'s in distances, angles and torsion angles; correlations between e.s.d.'s in cell parameters are only used when they are defined by crystal symmetry. An approximate (isotropic) treatment of cell e.s.d.'s is used for estimating e.s.d.'s involving l.s. planes.
Refinement. Refinement of *F*^2^ against ALL reflections. The weighted *R*-factor *wR* and goodness of fit *S* are based on *F*^2^, conventional *R*-factors *R* are based on *F*, with *F* set to zero for negative *F*^2^. The threshold expression of *F*^2^ > σ(*F*^2^) is used only for calculating *R*-factors(gt) *etc*. and is not relevant to the choice of reflections for refinement. *R*-factors based on *F*^2^ are statistically about twice as large as those based on *F*, and *R*- factors based on ALL data will be even larger.

### Fractional atomic coordinates and isotropic or equivalent isotropic displacement parameters (Å^2^)

**Table:**

	*x*	*y*	*z*	*U*_iso_*/*U*_eq_	
C1	0.5886 (2)	0.25153 (11)	0.23432 (10)	0.0172 (2)	
C2	0.7514 (2)	0.14590 (11)	0.34545 (10)	0.0201 (2)	
H2A	0.6664	0.1673	0.4289	0.024*	
H2B	0.9522	0.1560	0.3350	0.024*	
C3	0.7394 (2)	−0.01067 (12)	0.34647 (11)	0.0264 (3)	
H3A	0.8587	−0.0815	0.4150	0.032*	
H3B	0.5406	−0.0233	0.3673	0.032*	
C4	0.8456 (3)	−0.04206 (13)	0.21466 (12)	0.0290 (3)	
H4A	0.8225	−0.1407	0.2159	0.035*	
H4B	1.0513	−0.0415	0.1989	0.035*	
C5	0.6822 (2)	0.07141 (13)	0.10418 (11)	0.0272 (3)	
H5A	0.4801	0.0636	0.1148	0.033*	
H5B	0.7631	0.0511	0.0199	0.033*	
C6	0.6986 (2)	0.22686 (12)	0.10284 (10)	0.0217 (2)	
H6A	0.8983	0.2385	0.0843	0.026*	
H6B	0.5815	0.2992	0.0343	0.026*	
C7	−0.0539 (2)	0.51593 (11)	0.19148 (10)	0.0173 (2)	
C8	−0.1182 (2)	0.51675 (11)	0.33402 (10)	0.0188 (2)	
H8A	−0.1150	0.4156	0.3873	0.023*	
H8B	0.0320	0.5522	0.3629	0.023*	
C9	−0.3962 (2)	0.61033 (11)	0.35514 (10)	0.0190 (2)	
N1	0.36168 (17)	0.33947 (9)	0.26408 (8)	0.0176 (2)	
N2	0.19288 (17)	0.42828 (9)	0.16325 (8)	0.0183 (2)	
H1	0.227 (3)	0.4221 (14)	0.0801 (14)	0.022*	
N3	−0.61224 (19)	0.68227 (10)	0.37675 (9)	0.0251 (2)	
O1	−0.21717 (15)	0.59183 (8)	0.10701 (7)	0.0228 (2)	

### Atomic displacement parameters (Å^2^)

**Table:**

	*U*^11^	*U*^22^	*U*^33^	*U*^12^	*U*^13^	*U*^23^
C1	0.0182 (4)	0.0185 (5)	0.0157 (5)	−0.0059 (4)	−0.0009 (4)	−0.0038 (4)
C2	0.0207 (5)	0.0225 (5)	0.0153 (5)	−0.0025 (4)	−0.0019 (4)	−0.0038 (4)
C3	0.0310 (6)	0.0197 (5)	0.0229 (6)	−0.0032 (4)	0.0056 (5)	−0.0016 (4)
C4	0.0347 (6)	0.0210 (5)	0.0309 (6)	−0.0078 (4)	0.0109 (5)	−0.0102 (5)
C5	0.0217 (5)	0.0400 (7)	0.0266 (6)	−0.0090 (5)	0.0045 (4)	−0.0201 (5)
C6	0.0179 (5)	0.0282 (6)	0.0150 (5)	−0.0001 (4)	0.0000 (4)	−0.0036 (4)
C7	0.0189 (5)	0.0185 (5)	0.0151 (5)	−0.0058 (4)	0.0002 (4)	−0.0043 (4)
C8	0.0192 (5)	0.0217 (5)	0.0155 (5)	−0.0036 (4)	−0.0009 (4)	−0.0057 (4)
C9	0.0241 (5)	0.0199 (5)	0.0149 (5)	−0.0085 (4)	0.0000 (4)	−0.0050 (4)
N1	0.0196 (4)	0.0185 (4)	0.0142 (4)	−0.0041 (3)	−0.0031 (3)	−0.0027 (3)
N2	0.0196 (4)	0.0215 (5)	0.0118 (4)	−0.0018 (3)	−0.0011 (3)	−0.0034 (3)
N3	0.0253 (5)	0.0255 (5)	0.0240 (5)	−0.0037 (4)	0.0020 (4)	−0.0087 (4)
O1	0.0221 (4)	0.0263 (4)	0.0163 (4)	0.0009 (3)	−0.0027 (3)	−0.0043 (3)

### Geometric parameters (Å, º)

**Table:**

C1—N1	1.2845 (13)	C5—H5A	0.99
C1—C2	1.5032 (14)	C5—H5B	0.99
C1—C6	1.5036 (14)	C6—H6A	0.99
C2—C3	1.5371 (15)	C6—H6B	0.99
C2—H2A	0.99	C7—O1	1.2306 (12)
C2—H2B	0.99	C7—N2	1.3442 (13)
C3—C4	1.5269 (16)	C7—C8	1.5209 (14)
C3—H3A	0.99	C8—C9	1.4622 (14)
C3—H3B	0.99	C8—H8A	0.99
C4—C5	1.5273 (17)	C8—H8B	0.99
C4—H4A	0.99	C9—N3	1.1457 (13)
C4—H4B	0.99	N1—N2	1.3938 (12)
C5—C6	1.5314 (16)	N2—H1	0.900 (14)
			
N1—C1—C2	116.83 (9)	C4—C5—H5B	109.4
N1—C1—C6	128.63 (9)	C6—C5—H5B	109.4
C2—C1—C6	114.13 (8)	H5A—C5—H5B	108.0
C1—C2—C3	108.83 (9)	C1—C6—C5	108.10 (9)
C1—C2—H2A	109.9	C1—C6—H6A	110.1
C3—C2—H2A	109.9	C5—C6—H6A	110.1
C1—C2—H2B	109.9	C1—C6—H6B	110.1
C3—C2—H2B	109.9	C5—C6—H6B	110.1
H2A—C2—H2B	108.3	H6A—C6—H6B	108.4
C4—C3—C2	111.01 (9)	O1—C7—N2	122.06 (9)
C4—C3—H3A	109.4	O1—C7—C8	121.97 (9)
C2—C3—H3A	109.4	N2—C7—C8	115.97 (9)
C4—C3—H3B	109.4	C9—C8—C7	111.64 (8)
C2—C3—H3B	109.4	C9—C8—H8A	109.3
H3A—C3—H3B	108.0	C7—C8—H8A	109.3
C3—C4—C5	111.44 (9)	C9—C8—H8B	109.3
C3—C4—H4A	109.3	C7—C8—H8B	109.3
C5—C4—H4A	109.3	H8A—C8—H8B	108.0
C3—C4—H4B	109.3	N3—C9—C8	177.32 (11)
C5—C4—H4B	109.3	C1—N1—N2	117.65 (9)
H4A—C4—H4B	108.0	C7—N2—N1	119.30 (9)
C4—C5—C6	111.25 (9)	C7—N2—H1	116.5 (8)
C4—C5—H5A	109.4	N1—N2—H1	123.6 (8)
C6—C5—H5A	109.4		
			
N1—C1—C2—C3	114.56 (10)	O1—C7—C8—C9	3.02 (14)
C6—C1—C2—C3	−58.72 (11)	N2—C7—C8—C9	−177.51 (9)
C1—C2—C3—C4	54.68 (12)	C2—C1—N1—N2	−173.88 (8)
C2—C3—C4—C5	−54.91 (12)	C6—C1—N1—N2	−1.72 (16)
C3—C4—C5—C6	55.98 (12)	O1—C7—N2—N1	−176.79 (9)
N1—C1—C6—C5	−113.10 (12)	C8—C7—N2—N1	3.75 (13)
C2—C1—C6—C5	59.23 (11)	C1—N1—N2—C7	176.44 (9)
C4—C5—C6—C1	−56.13 (11)		

### Hydrogen-bond geometry (Å, º)

**Table:**

*D*—H···*A*	*D*—H	H···*A*	*D*···*A*	*D*—H···*A*
N2—H1···O1^i^	0.900 (14)	2.052 (15)	2.9399 (12)	168.4 (11)
C6—H6*B*···O1^i^	0.99	2.32	3.2736 (13)	161
C8—H8*B*···N3^ii^	0.99	2.41	3.3783 (14)	165

Symmetry codes: (i) −*x*, −*y*+1, −*z*; (ii) *x*+1, *y*, *z*.
