# Supplementary figures and images for: Crystal structure of 2-cyano-N′-(cyclo­hexyl­idene)acetohydrazide
Source: Acta Crystallogr Sect E Struct Rep Online. 2014 Aug 1;70(Pt 9):o886. doi: 10.1107/S1600536814009350 (PMC4186197; doi:10.1107/S1600536814009350)

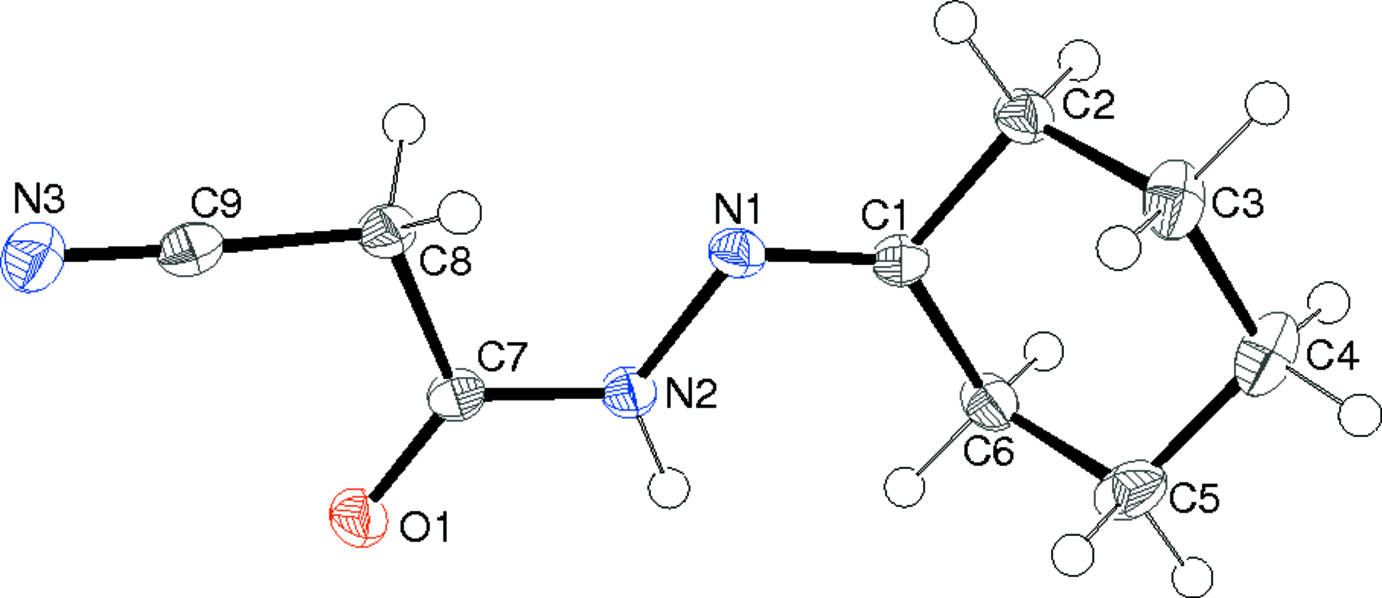

Supplement: Supplementary file 4 [file e-70-0o886-fig1.tif]

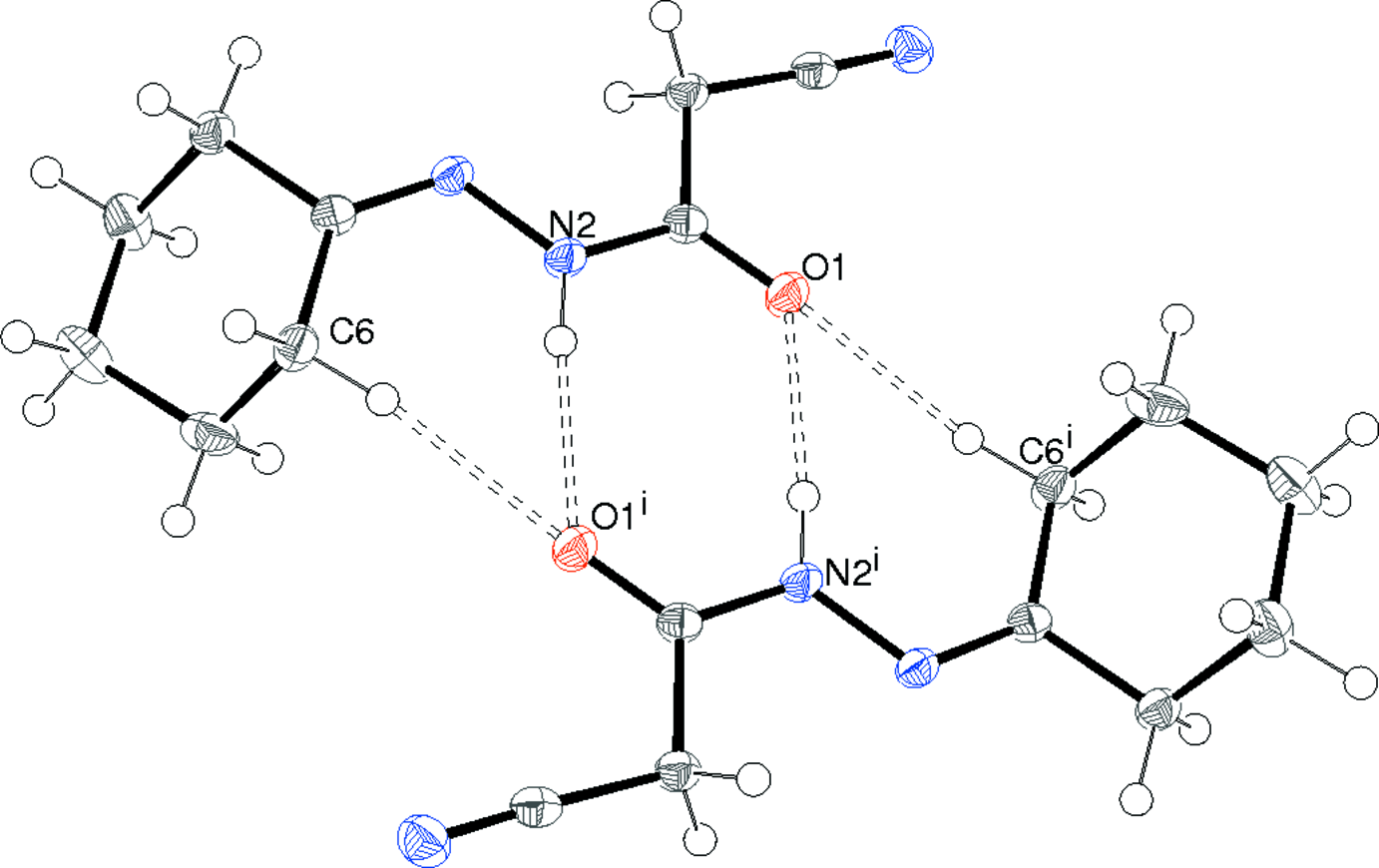

Supplement: Supplementary file 5 [file e-70-0o886-fig2.tif]

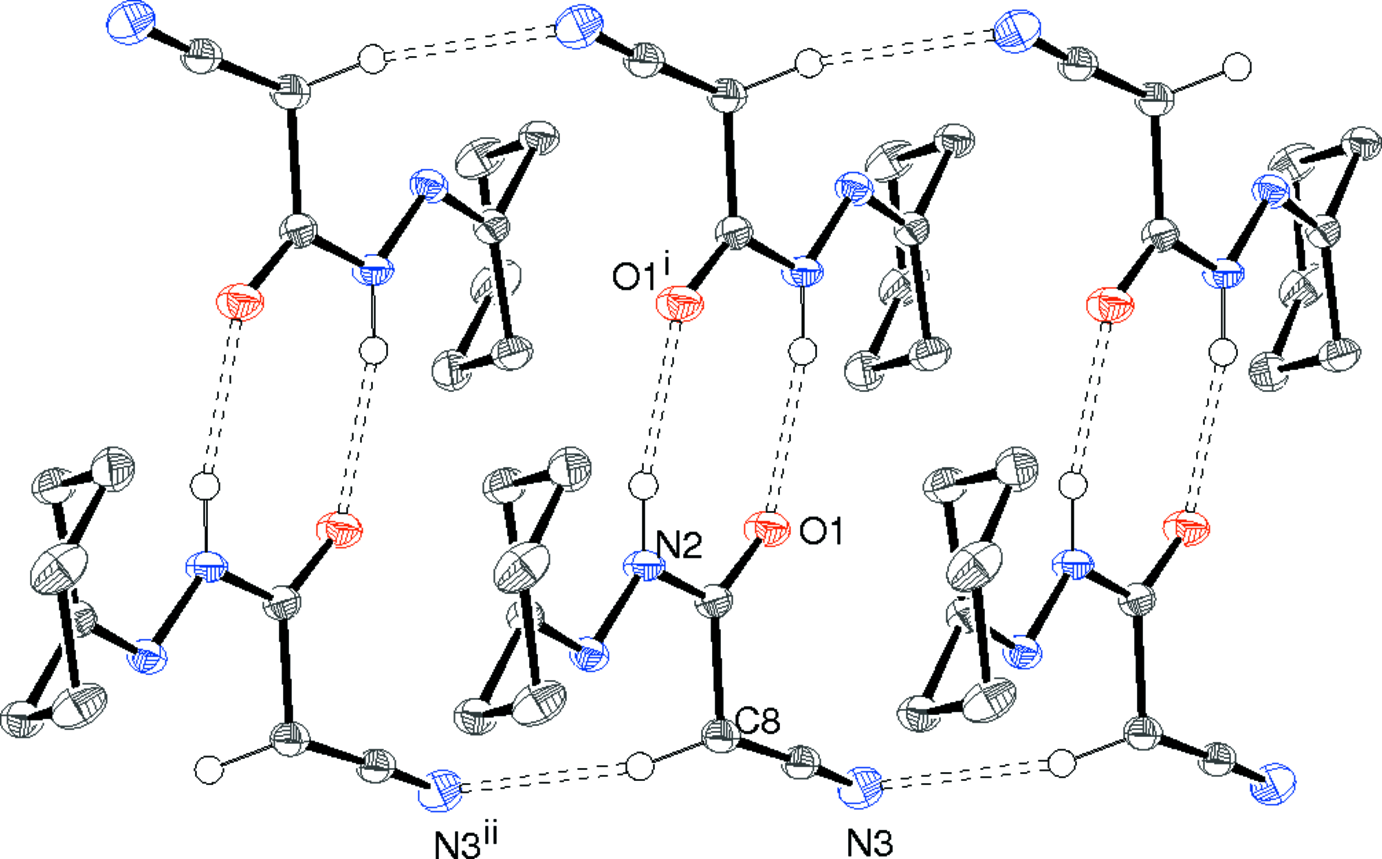

Supplement: Supplementary file 6 [file e-70-0o886-fig3.tif]
